# Mechanisms and Severity of Injuries in Infants and Children <2 Years: A Retrospective Analysis over 30 Years in a European Urban Level 1 Trauma Center

**DOI:** 10.3390/pediatric18010025

**Published:** 2026-02-05

**Authors:** Vanessa Groß, Anna Theresa Schauß, Lara Marie Bogensperger, Antonia Schwarz, Bikash Parajuli, Sanika Rapole, Janina M. Patsch, Notburga Payr, Kurt Payr, Stephan Payr

**Affiliations:** 1 Department of Trauma Surgery, University Clinic of Orthopedics and Trauma Surgery, Medical University of Vienna, 1090 Vienna, Austria; n11916964@students.meduniwien.ac.at (V.G.); n11817776@students.meduniwien.ac.at (A.T.S.); n11807478@students.meduniwien.ac.at (L.M.B.); n12220006@students.meduniwien.ac.at (A.S.); 2 Section of Pediatric Trauma Surgery, Department of Trauma Surgery, University Clinic of Orthopedics and Trauma Surgery, Medical University of Vienna, 1090 Vienna, Austria; 3 Department of Orthopedics and Traumatology, Dhulikhel Hospital, Kathmandu University, Kavre 45200, Nepal; bikash@kusms.edu.np; 4 Department of Pediatric Orthopedics, Sancheti Institute for Orthopedics and Rehabilitation, Pune 411005, India; drsanikarapole@gmail.com; 5 Department of Biomedical Imaging and Image-Guided Therapy, Division of General and Pediatric Radiology, Medical University of Vienna, 1090 Vienna, Austria; janina.patsch@meduniwien.ac.at; 6 Ordination Dr. Kurt Payr, Linzer Strasse 84, 4050 Traun, Austria

**Keywords:** epidemiology, infants, injury, type, mechanism

## Abstract

**Background/Objectives:** Injuries remain a major cause of childhood morbidity and mortality in Europe, despite improved prevention. Infants under one year are particularly vulnerable because of limited motor control and complete dependence on caregivers. Existing studies are often small or cover broad age ranges, limiting infant-specific insights. This study aims to provide a comprehensive overview of injury types, mechanisms, and treatments, focusing exclusively on infants aged zero to one year. **Methods:** This retrospective study analyzed 29,574 infants and children (<2 years) treated at a level 1 trauma department from 1993 to 2022. Primary data included main diagnosis, injury mechanism, and treatment. Injuries were classified by diagnosis and mechanism. Surgeries were categorized by procedure type. **Results:** Injury frequency increased with age. A total of 31.1% of cases occurred in infants (<12 months) and 68.9% in children (12–24 months). Head injuries were the most common trauma type (44%), particularly among infants (69.9%; children: 32.2%), while wounds (infants: 10.2%; children: 31.5%) and fractures (infants: 4.2%; children: 8.4%) were more frequent in children. Falls were the predominant mechanism (77.9%) across both groups. Most injuries were treated conservatively. A total of 228 surgical interventions were performed (0.8%), mainly for wounds (54.8%) and fractures (30.3%). **Conclusions:** This study shows that, even within the first two years of life, child development shapes both injury frequency and type. As mobility and independence increase, injuries rise, from predominantly head trauma among infants to a higher incidence of wounds and fractures in children. The majority of injuries were minor and managed conservatively.

## 1. Introduction

In 2015, a total of 13,173 children in Europe died because of injuries, corresponding to an approximate injury mortality rate of 37.6 per 100,000 [1]. Over the past years, both incidence and mortality of childhood injuries have declined [2], a trend that has been partly attributed to the implementation of prevention strategies [3,4]. These preventive efforts are often approached through three key domains: educating and training caregivers in emergency response, adapting the environment to reduce risks, and implementing legal frameworks that safeguard child protection [4]. Simultaneously, preventative recommendations for children must balance protection with freedom, to still ensure the development of both risk awareness and motor competence [5]. Furthermore, effective prevention must account for developmental differences. Child development varies markedly across age groups, with each stage defined by distinct milestones [6,7]. These stages are linked to specific vulnerabilities, defined by activity level, cognitive ability, understanding of safety rules, and personality traits [8]. The present study focuses on infants aged 0 to 1 year, a group characterized by predominantly reflex-based and biologically programmed movement patterns [7]. Jenni et al. describe the first six months of life as a phase of “motoric helplessness” [7]. During this period, infants are almost entirely dependent on parents or legal guardians in daily life, which shifts the primary responsibility for injury prevention to caregivers [9]. At the same time, this dependence offers caregivers significant opportunities to reduce injury risk through conscious preventive measures. This vulnerability and dependence of infants underline the need for a thorough understanding of injury mechanisms, circumstances, and treatment for effective prevention. Injury data should, therefore, be analyzed by developmental stage, as infants differ fundamentally from older children in behavior and motor skills [7].

Existing research is limited by small sample sizes [10,11], a narrow focus on specific injury types [11,12], or the inclusion of broad age ranges that may dilute age-specific insights [2,13]. To date, no large-scale epidemiological study has provided a comprehensive overview of injury mechanisms, affected body regions, and treatment strategies in infants aged zero to one year, despite the explained developmental vulnerabilities.

Therefore, the present study aims to provide a comprehensive epidemiological overview of injury mechanisms, affected body regions, and treatment in infants and children <2 years.

## 2. Materials and Methods

This retrospective, epidemiological study was approved by the Ethical Committee of the Medical University of Vienna (EC-Code: 2129/2022) on 11 April 2023 and conducted according to the Declaration of Helsinki and its latest amendments. Patients aged <2 years who were treated in the Department of Trauma Surgery at the Medical University of Vienna between January 1993 and December 2022 were included in the study. In total, 32,709 patients were screened, and of these, 3135 had to be discarded. The cohort comprises all emergency department visits, including those patients subsequently admitted to the hospital. Data were retrieved from the AKIM (AKH Information Management) database at the Department of Trauma Surgery of the Medical University of Vienna. The disclosed data included age, sex, primary diagnosis, injury mechanism, year of occurrence, and treatment (conservative/surgical).

### 2.1. Main Parameters

#### 2.1.1. Diagnosis

The study population was divided into two cohorts: infants, defined as individuals below one year of age (<12 months), and children, further defined as individuals between 12 and 24 months. Injuries were grouped into six main categories according to the primary diagnosis: head injury, wound, fracture, contusion, pulled elbow, and other injuries.

- The spectrum of head injuries included contusion and concussion, which are classified as mild traumatic brain injury (TBI). Additionally, severe TBI encompasses more severe injuries such as skull fracture and intracranial hemorrhage. Although concussions are classified as mild TBIs, they were presented separately. This classification reflects the practice at the hospital under investigation, wherein concussions are regarded as a distinct indication for inpatient admission for subsequent monitoring.
- Wounds included laceration, excoriation, cut, bite, puncture wound, and amputation.
- Fractures included fractures of the upper extremity, lower extremity, thoracic region, and epiphysiolysis.
- Other injuries included sprain, muscular hematoma, lesion, rupture, dislocation, and burns.

#### 2.1.2. Injury Mechanism

For infants, injury causes were grouped into seven categories: fall, injury associated with objects or persons, entrapment injury, traffic accident, documented third-party fault, birth-related trauma, and other events. For children, the classification was identical except for the absence of birth trauma, resulting in six categories. Injuries were also analyzed in relation to mechanisms.

#### 2.1.3. Surgery

Surgical interventions were classified into wound dressing, skull trepanation, exploration or revision surgery, fracture reduction, refixation, wound closure, fasciotomy, septic surgery, ring band splitting, amputation, osteosynthesis, and intracranial pressure catheter placement. Performed interventions were also described according to type of injury and mechanism.

### 2.2. Statistical Analysis

Descriptive data are reported for the entire patient cohort, including absolute frequencies (n) and relative frequencies (%). In order to provide an epidemiological overview, the parameters described above were included. Data processing and visualization were carried out with Microsoft Excel (Version 16.89.1, Microsoft Corp., Redmond, WA, USA).

## 3. Results

### 3.1. Demography

The study population comprised 29,574 infants and children. Of these, 31.1% (9210) were infants (<12 months), while 68.9% (20,364) were children (12–24 months). In the younger group, 54.5% were boys (5017) and 45.5% were girls (4193). In the older cohort, the distribution was comparable, with 55.9% male (11,386) and 44.1% female (8978).

### 3.2. Injuries

A comprehensive list of all injury categories is provided in Table 1. Furthermore, the frequency of the three most common injuries in the respective decades is listed in Table 2.

### 3.3. Head Injuries and Traumatic Brain Injuries

The overall incidence of head injuries (12,999; 44.0%) was evenly distributed between infants (6436; 49.5%) and children (6563; 50.5%). However, within the respective age cohorts, the proportion of head injuries was more than twice as high in infants (6436; 69.9%) compared with children (6563; 32.2%).

**Figure 1:**
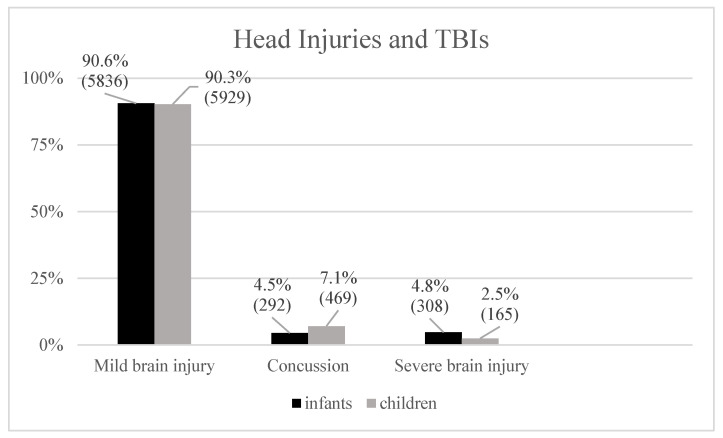
Compared frequencies (%) of head injuries in both cohorts.

Mild traumatic brain injuries (TBIs) such as contusions were documented within the age groups with 5836 (90.6%) infants and 5929 (90.3%) children (Figure 1). Less common TBIs, such as concussions, were more prevalent in the older cohort, with 469 (7.1%) children compared to 292 (4.5%) infants in the younger group. Severe TBIs, such as intracranial hemorrhages and skull fractures, occurred in 308 (4.8%) infants and 165 (2.5%) children. Out of 13 intracranial hemorrhages, 7 (0.1%) occurred in infants, and 6 (0,1%) occurred in children.

### 3.4. Wounds

The overall incidence of wounds was the second highest (7358; 24.9%), and wounds occurred predominantly in children (6423; 87.3%), with only 935 (12.7%) observed in infants. The relative frequency of wounds also differed between subgroups: approximately one-third of the children (6423; 31.5%) sustained a wound, compared with about 10.2% (935) of infants.

**Figure 2:**
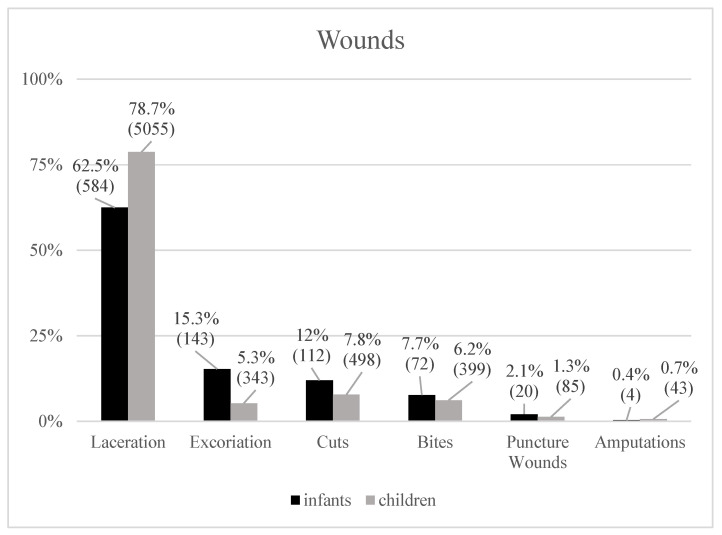
Compared frequencies (%) of wounds in both cohorts.

Lacerations were the most frequent within both age groups, affecting 584 (62.5%) infants and 5055 (78.7%) children (Figure 2). Excoriations ranked second among infants (143; 15.3%) but were relatively less common in children (343; 5.3%). Although cuts and bites were the second and third most common wounds among children (cuts: 498; 7.8%; bites: 399; 6.2%), relatively, percentagewise, these injuries occurred more often in the younger age cohort (cuts: 112; 12.0%; bites: 72; 7.7%).

### 3.5. Fractures

Overall, 2095 (7.1%) fractures (388; 4.2% within infants and 1707; 8.4% within children) were recorded.

**Figure 3:**
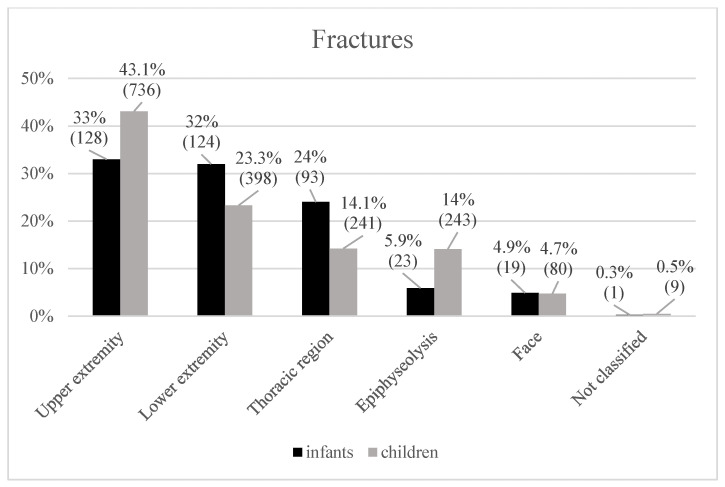
Compared frequencies (%) of fractures in both cohorts.

In infants, fractures of the upper and lower extremities occurred with similar frequency (upper: 128; 33%; lower: 124; 32%) (Figure 3). Within this age group, fractures in the humerus were the most common in the upper extremities (50 of 128; 39.1%). Femoral (66 of 124; 53.2%) and tibial fractures (35 of 124; 28.2%) predominated in the lower extremities. Thoracic fractures (93; 24%) mainly affected the clavicle and ribs (clavicle: 89, ribs: 23).

Children revealed more fractures of the upper extremity than the lower extremity (upper: 736; 43.1%; lower: 398; 1707). For the older age group, the pattern shifted toward more distal radial (247 of 736; 33.6%) and forearm (209 of 736; 28.4%) fractures in the upper extremities and tibial fractures (150 of 498; 30.1%) in the lower extremities. The children also had a lower proportion of thoracic fractures (241; 14.1%; clavicle: 239; rib: 1; vertebral: 1) than the infants.

### 3.6. Other and Potentially Life-Threatening Injuries

Additionally, 1317 (4.4%) other injuries occurred, with 268 (2.9%) in infants and 1049 (5.1%) in children. In both age groups, sprains were most common (infants: 92; 34.5%; children: 416; 39.7%), followed by lesions of the cheeks, lips, or teeth (infants: 54; 20.2%; children: 180; 17.2%) and muscular hematomas (infants: 45; 16.8%, children: 119; 11.3%).

Potentially life-threatening injuries among infants included one aortic rupture and one ruptured spleen. Among children, potentially life-threatening injuries included one hepatic rupture.

### 3.7. Injury Mechanism and Related Injuries

Generally, severe TBIs, including skull fractures and intracranial hemorrhage (epidural, subdural, subarachnoid) were observed less frequently and were mainly attributed to falls, falling objects, or collisions or occasionally following traffic accidents or due to third-party faults. Rare but medically noteworthy injuries, such as ruptures of the spleen, liver, or aorta, were exclusively linked to high-energy events like falls from significant heights (ranging from the 1st to 4th building floor) or traffic accidents.

Wounds typically followed falls, cuts, or bites but were not associated with life-threatening consequences.

Fractures were mostly linked to falls, with a smaller share following entrapment injuries or traffic accidents. The frequency of injury mechanisms in the overall patient group and in the different age groups is listed in Table 3, while the frequency of the four most common injury mechanisms within the respective decades is listed in Table 4. The frequency of resulting injuries according to injury mechanism is listed in Table 5.

### 3.8. Fall-Related Injuries

Falls were the predominant cause of injury in both groups, accounting for 23,018 cases (77.9%) overall, with nearly identical proportions in infants (7234; 78.5%) and children (15,784; 77.5%). Contusions of the head (mild TBIs) were the vast majority of fall-related injuries in infants (5215; 72.1% of fall-related injuries), whereas in children, it was about one-third (5566; 35.3%). Fall-related wounds and fractures occurred in larger proportions in children (wounds: 5200, 32.9%; fractures: 1453, 9.3%) than in infants (wounds: 551, 7.6%; fractures: 468, 6.5%). Severe outcomes within the entire patient collective included 10 fall-related intracranial hemorrhages (5 among each age group) and rare visceral injuries such as ruptures of the spleen (1 among children), liver (1 among infants), and aorta (1 among children).

### 3.9. Object/Person-Related Injuries

Object- or person-related injuries encompassed a range of mechanisms, including, for instance, being struck by objects or colliding with other individuals. Overall, this injury mechanism differed between age groups. In infants, injuries caused by objects or persons ranked second (888; 9.6%), while in children, this mechanism was relatively less common (1154; 5.7%). Object- or person-related injuries also revealed that contusions were most frequent in infants (530; 59.7%), with the majority affecting the head (411 of 530; 77.5%). Among children, pulled elbow was more prevalent (637; 55.2%). Contusions were less common (309; 26.8%) among children and more often involved the extremities (176 of 309; 57%) than the head (106 of 309; 34.3%).

### 3.10. Entrapment

Entrapment injuries usually occurred when body parts became trapped between objects, mainly furniture or doors. Overall, entrapment injuries were rare among infants (120; 1.3%) but occurred more than three times as often in children (805; 4%). In both age groups, entrapment injuries mostly led to contusions (infants: 68; 56.7%, children: 490; 61.6%). Wounds were more common in older infants (147; 18.5%) than in younger ones (18; 15%). Fractures were infrequent but slightly higher in children (70; 8.8%) compared to infants (9; 7.5%).

### 3.11. Traffic Accident

Traffic accidents mainly resulted in contusions (infants: 124; 83.2%, children: 113; 75.3%), with the majority involving the head (infants: 102; 82.3%, children: 91; 80.5%). Fractures occurred at similar rates in children (12; 8%) and in infants (8; 5.4%), with skull fractures predominating in infants (6 of 8; 75%) and extremity fractures in children (10 of 12; 83.3%). Wounds (infants: 6; 4%; children: 11; 7.3%) and concussions (infants: 7; 4.7%; children: 7; 4.6%) occurred at comparable rates among both age groups. Severe outcomes of traffic accidents were uncommon but included one subarachnoid hematoma (among children) and a ruptured spleen (among infants).

### 3.12. Documented Third-Party Fault

Contusions, also including muscular hematomas, were the most frequent injury (infants: 15; 71.4%, children: 10; 71.4%) due to documented third-party faults. Isolated cases included one concussion and an ear wound among the younger age group and one radial fracture and two wounds among the older age group.

### 3.13. Birth Trauma

The category of birth trauma naturally only affected the younger age group, with 20 patients (0.2%). 19 (95%) cases of birth traumas resulted in fractures (9 clavicular, 7 humeral, 3 femoral, and 2 tibial), while a single case (5%) involved a contusion of the head.

### 3.14. Other Injury Mechanisms

The total number of other injury mechanisms was 778 (8.4%) among infants and 2456 (12.1%) among children. In infants, the most common mechanism was play-related incidents (121; 15.6%), followed by bites (57; 7.3%) and cutting injuries (56; 7.2%), while cutting injuries (387; 15.8%) and furniture-related accidents (202; 8.2%) represented the next most common categories in children.

### 3.15. Surgery

In general, surgical interventions in the cohort were uncommon (228; 0.8% of 29,574 injuries), yet surgical intervention was documented three times as often in children (199; 1% of 20,364) compared to infants (29; 0.3% of 9210) (Table 6). In infants, surgeries were dominated by surgical wound management and explorations (10; 34.5%) and skull trepanations (4; 13.8%), whereas in children, the focus shifted toward exploration surgeries of wounds or fractures (66; 33.2%) and osteosyntheses (43; 21.6%). Fracture reduction maneuvers were performed rarely in both groups (infants: 4; 13.8%, children: 5; 2.5%). Wound closures through stitches and necessary refixations of structures were more frequent in children (31; 15.6% and 5; 2.5%) compared with infants (2; 6.9% for both surgery types).

Severe interventions were rare in both groups. Skull trepanations were documented in infants (4; 13.8%) and in children (3; 1.5%). Amputations (4; 2%) and intracranial pressure catheters (4; 2%) occurred only in the older cohort.

In the cohort of surgically treated patients with meticulously documented procedures (200; procedures categorized as “other” (28) were excluded), the following distribution was observed: 70.5% (141) received sedoanalgesia, 21.5% (43) underwent general anesthesia, and 8.0% (16) received a combination of plexus anesthesia and general anesthesia. Sedoanalgesia was predominantly used for explorations, wound closures or dressings, and reductions. In contrast, all patients undergoing trepanations (exclusively skull trepanations), fasciotomies, septic surgery, ring band splitting, implantation of intracranial pressure catheters, and amputations were treated under general anesthesia.

In the cohort of patients with surgically treated extremity fractures requiring osteosynthesis, the utilization of K-wires was a universal practice. The anesthetic techniques employed in this subgroup included sedoanalgesia in 8 patients, general anesthesia in 21 patients, and a combination of plexus and general anesthesia in 14 patients.

### 3.16. Surgically Treated Injuries

Across all surgically treated injuries (228; 100%), wounds (125; 54.8%) and fractures (69; 30.3%) were the leading reasons for surgery (Table 7). However, these 125 wounds represented only 1.7% of all wounds, which were mainly cuts (56) and amputations (32) followed by bites (14), lacerations (13), and puncture wounds (9).

Fractures required management in the operation room in 69 cases (3.4% of all fractures), predominantly in children (63; 91.3%) versus 6 cases (8.7%) in infants. The upper extremity (children: 61 of 69; 88.4%) was mostly involved. These upper extremity fractures mainly included the following types: humeral, forearm, and finger fractures.

Head injuries required surgical interventions (14; 0.1% of all head injuries), with a higher proportion in children (9; 64.3%) than in infants (5; 35.7%). Ten surgically treated skull fractures were equally distributed between the age groups. Surgically treated intracranial hemorrhages only occurred in four children.

### 3.17. Injury Mechanisms Leading to Surgery

Generally, falls were the leading cause of severe injuries leading to surgery (85; 37.3%) (Table 8). Among these, fractures (55) were the leading indication, followed by wounds (19), also including one rare rupture of the liver. Procedures after entrapment injuries were less frequent (36; 15.8%). When injuries were caused by an object or person, surgery was required in 18 (7.9%) cases, due to fractures (6), wounds (5), 4 amputations (3 total/1 subtotal), and septic complications (3) as the main indications. Four acute surgical intervention surgeries were necessary due to traffic accidents.

## 4. Discussion

This study highlights the frequency of injuries among infants and children younger than 2 years over a 29-year period (1993–2022). During this time, a total of 29,574 infants and children were injured, which equates to approximately 1000 injured patients per year. This large number of patients underscores the clinical and epidemiological relevance of the topic. Nevertheless, there is a notable lack of studies that systematically investigate the broader context of injuries in infants and children, including detailed analyses of injury subtypes and localization, underlying mechanisms, as well as the frequency and type of surgical interventions required.

In this study’s patient cohort, an increase in injuries was notable with advancing age. Of all injuries, 31.1% occurred in infants younger than 12 months, whereas 68.9% were observed in children aged 12–24 months. Similar findings were reported by Agran et al., who demonstrated a comparable increase in injuries among patients aged 0–24 months, with a peak incidence at 15 to 17 months [14]. This age group corresponds to the children in the present study. The developmental trajectory provides a plausible explanation for this trend: as children grow older, they gain greater mobility and independence from their caregivers [7,15]. However, despite being able to roll over, stand, or even take their first steps, infants and children at this stage still lack the cognitive capacity to recognize hazardous situations, leaving them particularly vulnerable to injuries [9,15].

The developmental stage as well as anatomical characteristics provide important context for understanding the pattern of injury types in this study [10,16,17,18]. In early life, the head is the most dominant anatomical feature and accounts for about one quarter of total body length [12,19]. This makes it especially vulnerable to external forces [12,19]. At the same time, infants and children lack sufficient muscular strength for adequate head and body control, which further limits their ability to buffer impacts [7]. This combination of anatomical and developmental factors is reflected in the presented findings, where 44% of all injuries were traumas to the head [7,19]. Other studies have reported even higher proportions: Beck et al. found head trauma in 63% of infants [16], while Cooray et al. observed head injuries in 85% of children under 12 years following falls [17]. In this present analysis, head injuries remained the most frequent category in both age groups. Interestingly, the rate was more than twice as high among infants (69.9%) compared with children (32.2%), suggesting the younger the infant or child, the more prone and the greater vulnerability to head trauma, which is different from general observations. Of the 12,999 head injuries, only 14 required neurosurgical intervention, indicating that head injuries are common, but severe cases needing surgery appear to be rare. Nevertheless, infants and children in their first two years of life face an elevated risk of head injuries, making prevention a central priority. Because they are fully dependent on their caregivers, effective prevention requires close supervision and a safe living environment. This includes designing homes in a safer way, for example, by installing handrails on staircases and using safety devices such as cupboard locks [9,20,21].

The other trauma types mentioned in this study were generally more common among children. These primarily included wounds and fractures. Children had approximately three times the rate of wounds (31.5% vs. 10.2%) and twice the rate of fractures (8.4% vs. 4.2%) compared to infants. This pattern can be explained by developmental differences, as children begin to walk independently and explore their environment more actively [7,15,22]. The greater frequency of wounds and fractures in this age group may also explain why the majority of surgical procedures within this study (87.3%) were performed in children. Notably, wounds and fractures were the injury types most frequently requiring surgical intervention. Nonetheless, the overall proportion of surgical interventions was low compared with the total number of injuries (228 vs. 29,574). This indicates that most cases in both age groups were not severe and could be managed conservatively. This finding also aligns with current pediatric traumatology practice, where conservative treatment is generally preferred, particularly for fractures [23].

Although most injuries were minor, it is important to consider the underlying mechanisms that most frequently lead to trauma in infants and children. Falls represented the most common injury mechanism in both age groups. In this study, 77.9% of all injuries were attributable to falls, with comparable proportions among infants and children (78.5% vs. 77.5%). This finding is consistent with previous reports in the literature [24,25,26]. Although falls frequently resulted in head injuries in both groups, there was a difference in the rates: 72.1% of falls led to at least mild head trauma in infants compared with 35.3% in children. This finding further shows the disproportionate impact of falls on head injuries in infants younger than 12 months, with Unni et al. also reporting greater severity of fall-related injuries in infants [27]. Addressing fall mechanisms, therefore, becomes an important aspect of prevention; however, the circumstances differ by age group. Unni et al. found that, among children, the most common cause of falls was from furniture [27]. In this group, close supervision combined with environmental safety measures, such as the use of safety equipment like changing tables with guardrails, seems to be an appropriate strategy [3,20]. By contrast, among infants, the predominant fall mechanism was caregivers accidentally dropping the child or tripping while carrying them [27]. For this group, prevention should focus on vigilant supervision and a safe home environment, keeping it free of tripping hazards [4,9,27]. The second most frequent injury mechanism involved contact with objects or other persons (2042). Injury patterns varied between age groups: infants were mainly affected by contusions (59.7%), whereas pulled elbow injuries predominated among children (55.2%). Yet again, this distinction likely reflects differences in daily handling and exposure. Infants, who are still mostly carried by their caregivers, are rarely subjected to the type of arm traction that causes radial head subluxation [7,15,28,29]. In contrast, children are more mobile, interact more independently, and are, therefore, more prone to situations where an arm is pulled, increasing the risk for this specific injury [15,28,29]. Traffic-related injuries provided an interesting contrast, as here both age groups were typically exposed under similar conditions (e.g., secured in a child car seat). In these situations, developmental differences may play a minor role. Therefore, the resulting injury patterns appeared less divergent between age groups than in other injury mechanisms. Contusions were the most frequent injury type in both groups, occurring with comparable rates (infants: 83.2%; children: 75.3%). Other resulting injury types were also similarly distributed between cohorts. Taken together, these findings highlight the critical influence of age and developmental stage on both injury mechanisms and injury types in early childhood. Even within the narrow window of the first two years of life, small age differences translate into changes in motor and cognitive abilities and, therefore, alter risk profiles [7,15].

As this study is retrospective in nature, it carries a risk of information loss. Complete and systematic data collection was ensured to minimize this limitation. Although the study was limited to a single institution, the setting was a high-volume level 1 trauma center, representing the largest facility in Austria and a major trauma center in Europe. Due to the substantial sample size of the patient population, the dataset can be considered adequately representative, although, as a single-center study, there is a possibility that it may overrepresent severe traumatic injuries and does not necessarily reflect population assumptions. Furthermore, as a computer search study (except for data on surgical treatment, which were necessary to extract manually during the review process), it is imperative to note that each new trauma is meticulously documented, and as such, it is not feasible to provide a commentary on a patient who has repeatedly presented with a new injury on each occasion. Authors also regret to inform that specific data or comments regarding non-accidental cases cannot be provided due to organizational and data privacy restrictions. Generally, it is not possible to obtain official confirmation of non-accidental trauma through a computer search. This confirmation must be manually obtained, which is a nearly impossible task for this exceptionally large cohort. The terminology used in this manuscript—“third-party fault”—refers to injuries sustained in accidents involving third parties. However, it is important to note that authors are unable to categorize these injuries as non-accidental. Furthermore, it should be noted that the extensive sample size is also the reason for the focus on the limited number of parameters presented. The results of the present study provide a solid foundation for future research concerning injury patterns, treatment approaches, and underlying mechanisms in infants and children.

## 5. Conclusions

This study demonstrates that, even within the narrow age range of infants (<12 months) and children (12–24 months), developmental progression influences both the frequency and nature of injuries. As mobility and independence increase, the number of injuries rises. This is reflected in the distribution of injury types, with head injuries predominating overall, particularly among infants. In addition, the incidence of wounds and fractures increases in children. Falls remained the leading cause of trauma in both groups. The majority of injuries were minor and managed conservatively.

## Figures and Tables

**Table 1:** Frequency of injuries in both age groups.

Injuries	Infants Abs. | Column % | Row %	Children Abs. | Column % | Row %	Total Abs. | Column % | Row %
Head injury	6436 | 69.9% | 49.5%	6563 | 32.2% | 50.5%	12,999 | 44% | 100%
Wound	935 | 10.2% | 12.7%	6423 | 31.5% | 87.3%	7358 | 24.9% | 100%
Contusion	861 | 9.3% | 21%	3241 | 15.9% | 79%	4102 | 24.9% | 100%
Fracture	388 | 4.2% | 18.5%	1707 | 8.4% | 81.5%	2095 | 13.9% | 100%
Pulled elbow	322 | 3.5% | 18.9%	1381 | 6.8% | 81.1%	1703 | 5.8% | 100%
Other	268 | 2.9% | 20.3%	1049 | 5.1% | 79.7%	1317 | 4.4% | 100%
Total	9210 | 100% | 31.1%	20,364 | 100% | 68.9%	29,574 | 100% | 100%

**Table 2:** Most common injuries over the decades for both groups and overall.

Injuries over the Decades	1993–2002 Infant | Child | Total	2003–2012 Infant | Child | Total	2013–2022 Infant | Child | Total	Overall Infant | Child | Total
Head injury	8976 | 931 | 1827	2249 | 2354 | 4603	3291 | 3278 | 6569	6439 | 6563 | 12,999
Wound	238 | 1619 | 1857	349 | 2472 | 2821	348 | 2332 | 2680	935 | 6423 | 7358
Contusion	151 | 778 | 929	309 | 1219 | 1528	401 | 1244 | 1645	861 | 3241 | 4102

**Table 3:** Frequency of injury mechanisms.

Mechanism	Infants Abs. | Column % | Row %	Children Abs. | Column % | Row %	Total Abs. | Column % | Row %
Fall	7234 | 78.5% | 31.4%	15,784 | 77.5% | 68.6%	23,018 | 77.9% | 100%
Injury by object/person	888 | 9.6% | 43.5%	1154 | 5.7% | 56.6%	2042 | 6.9% | 100%
Entrapment	120 | 1.3% | 13.0%	805 | 4% | 87.0%	925 | 3.1% | 100%
Traffic accident	149 | 1.6% | 49.7%	151 | 0.7% | 50.3%	300 | 1.0% | 100%
Documented third-party fault	21 | 0.2% | 60%	14 | 0.1% | 40%	35 | 0.1% | 100%
Birth trauma	20 | 0.2% | 100%	- | 0% | 0%	20 | 0.07% | 100%
Other	778 | 8.4% | 24.1%	2456 | 12.1% | 75.9%	3234 | 10.9% | 100%
Total	9210 | 100% | 31.1%	20,364 | 100% | 68.9%	29,574 | 100.0% | 100%

**Table 4:** Most common injury mechanisms over the decades for both groups and overall.

Injury Mechanisms over the Decades	1993–2002 Infant | Child | Total	2003–2012 Infant | Child | Total	2013–2022 Infant | Child | Total	Overall Infant | Child | Total
Fall	1100 | 3167 | 4267	2583 | 5778 | 8361	3551 | 6839 | 10,390	7234 | 15,784 | 23,018
Injury by object/person	119 | 285 | 404	256 | 441 | 697	513 | 428 | 941	888 | 1154 | 2042
Entrapment	28 | 210 | 238	39 | 298 | 337	53 | 297 | 350	120 |805 | 925
Traffic accident	50 | 40 | 90	66 | 58 | 124	33 | 53 | 86	149 | 151 | 300

**Table 5:** Frequencies of injury mechanisms and the resulting injury types in each age cohort and the entire patient collective.

Fall	Infants Abs. | Column % | Row %	Children Abs. | Column % | Row %	Total Abs. | Column % | Row %
Mild TBI	5215 | 72.1% | 48.4%	5566 | 35.3% | 51.6%	10,781 | 46.8% | 100%
Wound	551 | 7.6% | 9.6%	5200 | 32.9% | 90.4%	5751 | 25% | 100%
Contusion	544 | 7.5% | 21%	2046 | 13% | 79%	2590 | 11.3% | 100%
Fracture	468 | 6.5% | 24.4%	1453 | 9.3% | 75.6%	1921 | 8.3% | 100%
Concussion	257 | 3.6% | 37%	437 | 2.8% | 63%	694 | 3% | 100%
Pulled elbow	50 | 0.7% | 10.3%	434 | 2.7% | 89.7%	484 | 2.1% | 100%
Sprain	33 | 0.5% | 16.1%	172| 1.1% | 83.9%	205| 0.9% | 100%
Hematoma	28 | 0.4% | 24.1%	88 | 0.6% | 75.9%	116 | 0.5% | 100%
Intracranial hemorrhage	5 | 0.01% | 50%	5 | 0.03% | 50%	10 | 0.04% | 100%
Other	83 | 1.1% | 17.8%	383 | 2.4% | 82.2%	466 | 2% | 100%
Total	7234 | 100% | 33%	15,784 | 100% | 67%	23,018 | 100% | 100%
**Injury by object/person**			
Contusion	530 | 59.7% | 63.2%	309 | 26.8% | 36.8%	839 | 41.1% | 100%
Wound	150 | 16.9% | 54.7%	124 | 10.7% | 45.3%	274 | 13.4% | 100%
Fracture	30 | 3.4% | 40%	45 | 3.9% | 60%	75 | 3.7% | 100%
Concussion	15 | 1.7% | 65.2%	8 | 0.7% | 34.8%	23 | 1.1% | 100%
Pulled elbow	134 | 15.1% | 17.4%	637 | 55.2% | 82.6%	771 | 37.8% | 100%
Hematoma	4 | 0.5% | 20%	16 | 1.4% | 80%	20 | 1% | 100%
Other	25 | 2.8% | 62.5%	15 | 1.3% | 37.5%	40 | 2% | 100%
Total	888 | 100% | 43.5%	1154 | 100% | 56.5%	2042 | 100% | 100%
**Entrapment**			
Contusion	68 | 56.7% | 12.2%	490| 61.6% | 87.8%	558| 60.9% | 100%
Wound	18 | 15% | 10.9%	147 | 18.5% | 89.1%	165 | 18% | 100%
Fracture	9 | 7.5% | 11.4%	70 | 8.8% | 88.6%	79 | 8.6% | 100%
Other	25 | 20.8% | 21.9%	89 | 11.2% | 78.1%	114 | 12.4% | 100%
Total	120 | 100% | 13.1%	796 | 100% | 86.9%	916 | 100% | 100%
**Traffic accident**			
Contusion	124 | 83.2% | 52.3%	113| 75.3% | 47.7%	237| 79.3% | 100%
Wound	6 | 4% | 35.3%	11 | 7.3% | 64.7%	17 | 5.7% | 100%
Fracture	8 | 5.4% | 40%	12 | 8% | 60%	20 | 6.7% | 100%
Concussion	7 | 4.7% | 50%	7 | 4.7% | 50%	14 | 4.7% | 100%
Other	4 | 2.7% | 36.4%	7 | 4.7% | 63.6%	11 | 3.7% | 100%
Total	149 | 100% | 49.8%	150 | 100% | 50.2%	299 | 100% | 100%
**Documented third-party fault**			
Contusion	15 | 71.4% | 62.5%	9 | 64.3% | 37.5%	24 | 68.6% | 100%
Wound	1 | 4.7% | 33.3%	2 | 14.3% | 66.7%	3 | 8.6% | 100%
Fracture	0 | 0% | 0%	1 | 7.1% | 100%	1 | 2.9% | 100%
Concussion	1 | 4.7% | 100.0%	0 | 0% | 0%	1 | 2.9% | 100%
Hematoma	4 | 19% | 66.7%	2 | 14.3% | 33.3%	6 | 17.1% | 100%
Total	21 | 100% | 60%	14 | 100% | 40%	35 | 100% | 100%

**Table 6:** Frequency of surgery types in each age cohort and the entire patient collective.

Type of Surgery	Infants Abs. | Column % | Row %	Children Abs. | Column % | Row %	Total Abs. | Column % | Row %
Exploration	10 | 34.5% | 13.2%	66 | 33.2% | 86.8%	76 | 33.3% | 100%
Osteosynthesis	0 | 0% | 0%	43 | 21.6% | 100%	43 | 18.9 | 100%
Wound closure	2 | 6.9% | 6.1%	31 | 15.6% | 93.9%	33 | 14.5% | 100%
Wound dressing	3 | 10.3% | 23.1%	10 | 5% | 76.9%	13 | 5.7% | 100%
Reduction	4 | 13.8% | 44.4%	5 | 2.5% | 55.6%	9 | 3.9% | 100%
Refixation	2 | 6.9% | 28.6%	5 | 2.5% | 71.4%	7 | 3.1% | 100%
Trepanation	4 | 13.8% | 56.1%	3 | 1.5% | 42.9%	7 | 3.1% | 100%
Fasciotomy	1 | 3.4% | 50%	1 | 0.5% | 50%	2 | 0.9% | 100%
Septic surgery	1 | 3.4% | 100%	0 | 0% | 0%	1 | 0.4% | 100%
Ring band splitting	1 | 3.4% | 100%	0 | 0% | 0%	1 | 0.4% | 100%
Intracranial pressure catheter	0 | 0% | 0%	4 | 2% | 100%	4 | 1.8% | 100%
Amputation	0 | 0% | 0%	4 | 2% | 100%	4 | 1.8% | 100%
Other	1 | 3.4% | 3.6%	27 | 13.5% | 96.4%	28 | 12.3% | 100%
Total	29 | 100% | 12.7%	199 | 100% | 87.3%	228 | 100%| 100%

**Table 7:** Frequency of surgery types in each age cohort and the whole patient collective.

Injury Requiring Surgery	Infants Abs. | Column % | Row %	Children Abs. | Column % | Row %	Total Abs. | Column % | Row %
Traumatic brain injury	5 | 17.2% | 35.7%	9 | 4.5% | 64.3%	14 | 6.1% | 100%
Wound	14 | 48.3% | 11.2%	111 | 55.8% | 88.8%	125 | 54.8% | 100%
Fracture	6 | 20.7% | 8.7%	63 | 31.7% | 91.3%	69 | 30.3% | 100%
Contusion	1 | 3.4% | 20%	4 | 2% | 80%	5 | 2.2% | 100%
Other	3 | 10.3% | 20%	12 | 6% | 80%	15 | 6.6% | 100%
Total	29 | 100% | 12.7%	199 | 100% | 87.3%	228 | 100% | 100%

**Table 8:** Frequency of injury mechanisms leading to surgery in each age cohort and the whole patient collective.

Injury Mechanism Leading to Surgery	Infants Abs. | Column % | Row %	Children Abs. | Column % | Row %	Total Abs. | Column % | Row %
Fall	10 | 34.5% | 11.8%	75 | 37.7% | 88.2%	85 | 37.3% | 100%
Entrapment	5 | 17.2% | 13.9%	31 | 15.6% | 86.1%	36 | 15.8% | 100%
Injury by object/person	2 | 6.9% | 11.1%	16 | 8.1% | 88.9%	18 | 7.9% | 100%
Traffic accident	0 | 0% | 0%	4 | 2% | 100%	4 | 1.8% | 100%
Other	9 | 31% | 14.3%	54 | 27.1% | 85.7%	63 | 27.6% | 100%
Not specified	3 | 10.3% | 13.7%	19 | 9.5% | 86.4%	22 | 9.6% | 100%
Total	29 | 100% | 12.7%	199 | 100% | 87.3%	228 | 100% | 87.3%

## Data Availability

The datasets generated and/or analyzed in the current study are not publicly available due to data privacy but are available from the corresponding author on reasonable request.
